# A Half Century of Research on Childhood and Adolescent Depression: Science Mapping the Literature, 1970 to 2019

**DOI:** 10.3390/ijerph18189524

**Published:** 2021-09-09

**Authors:** Mi Zhou, Biyu Bian, Weiming Zhu, Li Huang

**Affiliations:** College of Economics and Management, Shenyang Agricultural University, Shenyang 110866, Liaoning, China; zhoumi2011@syau.edu.cn (M.Z.); 2019220618@stu.syau.edu.cn (B.B.); 2020200182@stu.syau.edu.cn (W.Z.)

**Keywords:** depression, children, adolescents, science mapping, bibliometric analysis

## Abstract

In order to gain an in-depth understanding of research about childhood and adolescent depression, this article analyzes the scale, development, and geographic distribution of the literature in the field based on 8491 articles extracted from the Web of Science Core database. Using citation analysis, this article identifies influential journals, scholars, and documents in this field. The study found that in the past 15 years, the number of documents has increased significantly and geographical diversity has also increased. Most of the highly influential literature relates to depression inventories. Using keyword co-occurrence analysis, this article also identified three key research topics focusing on (a) child and adolescent depression symptoms and prevalence, (b) parental depression and child behavioral or emotional problems, and (c) childhood abuse and depression. This study uses ‘science mapping’ as a means to provide a better understanding of research trends about childhood and adolescent depression that have emerged over the past half century, and may serve as reference for future research.

## 1. Introduction

Depression is defined as a cluster of specific symptoms with associated impairment. The clinical and diagnostic features of the disorder are broadly similar in adolescents and adults [1,2,3]. The first article on depression in children and adolescents was published in 1934 by Schumacher. In his paper, he stated that depression had serious and long-lasting effects on mental health in both children and adults. Morbidity rates had become very high, and it was time for constructive work in the field of mental healthcare [4]. Before the 1970s, child psychiatry and psychology literature contained relatively few references to depression. Discussion of depression in children centered around the question of whether or not it existed [5]. Depression in children began to attract attention from researchers after the 1970s [6,7]. Early studies discussed the nature and characteristics of depression in childhood [6,8], the classification of childhood depression [9,10], the recognition of depressive disorders in children [11], and methods of measuring childhood depression [12,13]. In general, before the 1980s, studies on childhood depression still lacked using methodological techniques such as systematic rating scales and structured interviews for children analogous to those that have been developed for adults [14]. Since 1980s, more measurement scales were developed and studies of depression in children became more common.

Some empirical and qualitative reviews by experts have offered an overview of childhood and adolescent depression research, providing qualitative reviews of research on depression in children and adolescents from multiple perspectives, including epidemiology, family factors, individual characteristics, and treatment [15,16,17,18]. However, it is difficult to effectively organize, summarize, and quantitatively analyze the development of a specific field among a large number of studies on a large time scale in traditional review articles. Bibliometric analysis can effectively describe the knowledge status, features, and trends in a certain discipline and help people who are interested in but unfamiliar with this field to quickly grasp its basic status. This technique has been widely used to measure the performance of various disciplines [19,20]. Additionally, knowledge graphs combine information visualization technology with traditional scientometrics citation analysis to visually display the knowledge of a subject or field through data mining, information processing, scientific measurement, and graphic drawing [21,22].

The purpose of this paper is to gain an empirically-based perspective on the evolution of the childhood and adolescent depression knowledge base over a period of 50 years. We address the following three questions: (1) What is the growth trajectory and geographic distribution of the literature about childhood and adolescent depression published between 1970 and 2019? (2) Which journals, authors and articles were most influential during this period? (3) What topics have attracted the most attention from scholars in this field over the past 50 years?

Specifically, this paper examines the literature on childhood depression included in Web of Science (WoS) core database from 1970 to 2019 and conducts a simple statistical analysis to obtain the basic development law and regional distribution of the literature on childhood depression. This paper also uses descriptive statistics, citation analysis and co-citation analysis to display the productive journals, core authors, and important literature in the field of childhood depression research, and makes a visual analysis of the co-occurrence network of key words in studies on childhood depression with the help of VOSviewer software. Additionally, this paper combines analysis of the visualization results and the literature to dig out the knowledge base and research hotspots in the field of childhood depression. Finally, the paper summarizes the results of the bibliometric analysis and gives suggestions for future research.

## 2. Methodology

The methods that used for bibliometric analysis include descriptive statistics, citation analysis, co-citation analysis, and co-occurrence analysis [23]. Scholars have used citation analysis to identify well-known authors, documents and journals in a certain field of knowledge [24,25,26]. Co-citation analysis can ‘capture’ some important documents by mining the reference list of documents in a certain database. It therefore provides a broader method of measuring academic influence than traditional citation analysis. This article uses MS Excel and WoS analysis tools to perform descriptive statistical analysis of trends in the literature (such as the number of articles classified by country, author, and journal). This article also uses ArcGIS to create a heat map showing the geographic distribution of documents. It also uses citation analysis to determine the extent to which authors and documents in the database had been cited. Document co-citation analysis (DCA) is used to identify influential articles. Finally, this study uses the freely accessible text mining software VOSviewer to analyze and visualize the literature on child and adolescent depression from the perspectives of authors, citations, and keywords.

Data collection is a crucial part of bibliometric analysis. The academic database Web of Science (WoS) covers nearly 150 research disciplines and contains over 12,000 influential academic journals that are widely recognized by the international academic community [27]. The literature used in this study comes from the “Science Citation Index Extension (SCI-E)” and “Social Science Citation Index (SSCI)” databases of the “Web of Science Core Collection”. On 30 August 2021, an advanced search was performed in WoS using the following search string: TI = ((depression AND child*) or (depressive symptom* AND child*) or (depressive disorder* AND child*) or (psychological distress* AND child*) or (mood disorder* AND child*) or (depression AND adolescen*) or (depressive symptom* AND adolescen*) or (depressive disorder* AND adolescen*) or (psychological distress* AND adolescen*) or (mood disorder* AND adolescen*)). The topic field retrieval, which includes the fields “Title”, “Abstract”, and “Keywords”, was not used. This allows the study to avoid the occurrence of non-related results. The timespan was set from 1970 to 2019. The retrieved document records provided several fields, including title, author, abstract, source publication, citations, and references.

## 3. Results

### 3.1. Volume, Growth Trajectory, and Geographic Distribution of the Literature

The sample in this study is comprised of 8491 articles by 24,950 authors affiliated with 4510 institutions in 113 countries. These articles were published in 1240 journals and included 175,363 cited references.

**Figure 1:**
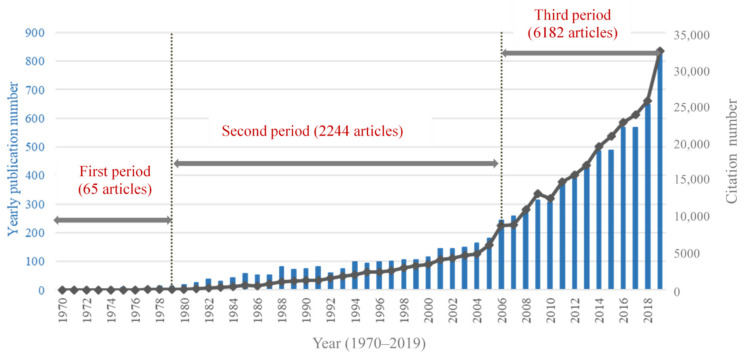
Trends in the quantity of articles identified by Web of Science (WoS) that are related to childhood and adolescent depression and published between 1970 and 2019.

The number of papers published can provide an important reference for the study of the development characteristics and laws of a certain field during a certain period. Figure 1 illustrates the annual number and the citation volume of articles on childhood and adolescent depression. In general, the number of articles on child and adolescent depression has increased significantly from the initial 3 in 1970 to 824 in 2019. However, as can be seen from the figure, the number of annual articles published did not increase yearly from 1970–2019. Meanwhile, 2019 was the year with the most published studies of childhood and adolescent depression (824 articles published).

We compared the annual publication volume (Figure 1), dividing the years into the following three stages: 1970–1979, 1980–2005, and 2006–2019. Only a handful of studies were published in each year before 1980 (an average of 6.5 articles per year from 1970–1979). However, an upward trend can be observed after 1980 (with an average of 86.3 articles per year from 1980–2005). The number of published articles exceeded 200 for the first time in 2006, and has continued to increase significantly (with an average of 441.57 articles per year from 2006–2019).

**Figure 2:**
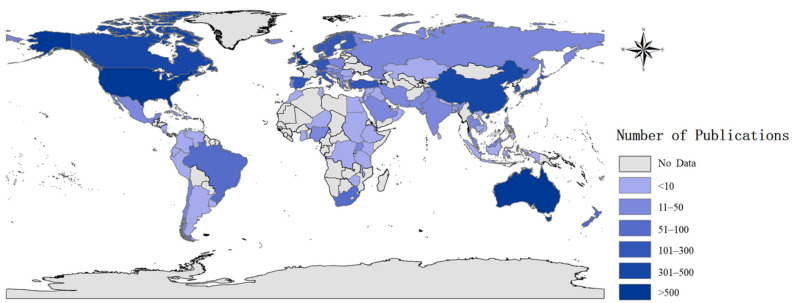
Global distribution of the childhood and adolescent depression literature, 1970 to 2019.

Figure 2 shows the geographic distribution of articles published since 1970 on childhood and adolescent depression. Darker colors signify higher rates of publication. The map shows the dominance of the United States, which accounts for nearly half of the documents in the database. Among the countries that published more than 100 articles, there are two North American countries, eight European countries, four Asian countries, and Australia. Articles about childhood and adolescent depression are mainly concentrated in the United States, England, Canada, Australia, China, Netherlands, and Germany.

### 3.2. Influential Journals, Authors, and Documents

#### 3.2.1. Journal Analysis

There were 1240 journals that published studies on depression in children and adolescents between 1970 and 2019. Table 1 lists the top ten journals in terms of publication volume. The Journal of Affective Disorders (352) is the most active journal, followed by Journal of The American Academy of Child and Adolescent Psychiatry (299), Journal of Abnormal Child Psychology (240), and Journal of Youth and Adolescence (163). Articles in the field of childhood and adolescent depression have been published primarily in these major journals. Specifically, the journals in Table 1 (accounting for less than1% of 1240 journals) published a total of 1759 articles, accounting for 20.7% of the total 8491 articles.

The impact factor (IF) and H-index of journals measure their value according to their role and status in scientific communication. Among the top-10 most productive journals, Journal of The American Academy of Child and Adolescent Psychiatry is the most influential in terms of IF and H-index (10.606 and 95), followed by Journal of Child Psychology and Psychiatry (9.917 and 55), and Journal of Abnormal Child Psychology (8.756 and 60). As shown in Table 1, each journal belongs to at least one of the following categories: Clinical Neurology, Psychiatry, Psychology Developmental, Psychology Clinical, Family Studies, Psychology Social, Social Work, or Psychology. Therefore, we can see that the study of childhood and adolescent depression is mainly concentrated in the fields of psychology, sociology and psychiatry.

#### 3.2.2. Author and Co-Authorship Analysis

“Author-based studies” have been one of the most important contributions of bibliometrics [28]. In practice, scholars identify prolific scholars based on the number of published papers and evaluate the academic influence based on the number of citations an author receives. The number of authors in the original download data is 25,569. Since there are sometimes multiple ways to write an author’s name (such as Kovacs, Maria and Kovacs, M. etc.), it is necessary to replace the author’s name while merging the information. With the help of the auxiliary function of the VOSviewer software (as a free piece of software, VOSviewer can be downloaded from the website https://www.vosviewer.com/download (accessed on 11 October 2020)), this study was carried out by two authors to ensure data accuracy. The final number of authors was 24,950. Table 2 lists the top 20 scholars by the number of citations and articles. Meanwhile, the number of average citations and H-index are analyzed to identify the most influential authors in the field.

It can be found that Seeley, JR, from Oregon Research Institute (USA), is the author with the most citations (8258) and the largest number of articles on childhood and adolescent depression (50). It is noteworthy that 19 of the 20 authors in Table 2 are from the United States. With regards to average number of citations, it can be found that Lewinsohn, PM, who is also from Oregon Research Institute, had made the greatest contribution to the field. Of the 20 authors, 11 have been active in the area of childhood depression for more than 30 years and 19 have been active for over 20 years. There are 17 authors who are still engaged in this research (meaning that they have published new articles in the field since 2016).

**Figure 3:**
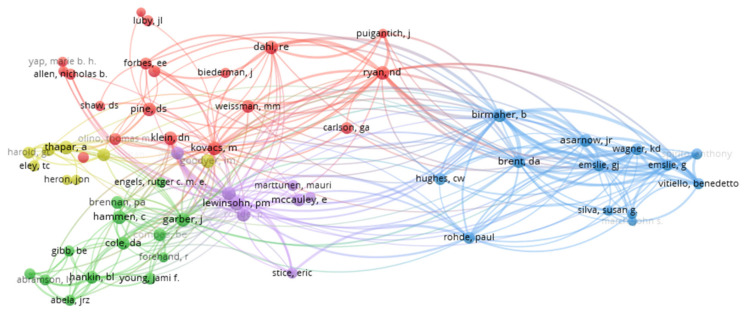
The co-authorship network for studies of child and adolescent depression, from 1970 to 2019 (threshold 20 articles, display 61 authors).

Here, we use co-author network analysis to highlight author cooperation in the study of childhood and adolescent depression. The minimum number of documents for an author was set as 20. Of the 24,950 authors, 61 met this threshold. The clustering function of VOS viewer software divides these authors into five categories and each cluster is marked with a different color. As shown in Figure 3, Garber, J contributes to the most documents with others (links = 59), followed by Klein, DN (links = 58), Kovacs, M (links = 57), and Emslie, GJ (links = 57).

#### 3.2.3. Citation and Co-Citation Analysis of Documents

In this section, we will focus on analyzing the most highly cited and co-cited documents. A total of 20 of the articles published from 1970–2019 have 570 or more citations (see Table 3). These highly-cited documents were published between 1981 and 2012. Six authors in Table 2 appear among the authors of the highly-cited articles listed in Table 3, and 7 of the 20 articles listed in Table 3 were authored (or co-authored) by scholars listed in Table 2.

Most of these highly-cited articles were concerned with “depression inventory” [29,30,31,32,33,34,35,36]; There have also been many studies of “childhood abuse or trauma and depression” and “adverse childhood experiences and depression” [37,38,39,40]. In addition, studies on the symptoms, characteristics, prevalence, and treatment of depression in children and adolescents also appear in Table 3.

**Table 3:** Ranking of the 20 most highly cited articles, 1970 to 2019.

Rank	Document	Topic	Citations ^a^
1	Kovacs, M. (1985) [30]. The Children’s Depression Inventory (CDI)	Depression Inventory	2432
2	Kovacs, M. (1981) [29]. Rating-scales to assess depression in school-aged children	Depression Inventory	1604
3	Lewinsohn, P.M., Hops, H., Roberts, R.E., Seeley, J.R., Andrews, J.A. (1993) [41]. Adolescent Psychopathology. 1. Prevalence and Incidence of Depression and Other DSM-III-R Disorders in High-School-Students	Depression Prevalence	1243
4	Radloff, L.S. (1991) [31]. The Use of The Center for Epidemiologic Studies Depression Scale in Adolescents and Young-Adults	Depression Inventory	1236
5	Chapman, D.P., Whitfield, C.L., Felitti, V.J., Dube, S.R., Edwards, V.J., Anda, R.F. (2004) [37]. Adverse childhood experiences and the risk of depressive disorders in adulthood	Childhood Abuse and Depressive Disorders	1153
6	Pine, D.S., Cohen, P., Gurley, D., Brook, J. (1998) [42]. The risk for early-adulthood anxiety and depressive disorders in adolescents with anxiety and depressive disorders	Depression Risk	1148
7	Angold, A., Costello, E.J., Messer, S.C., Pickles, A. (1995a) [34]. Development of a short questionnaire for use in epidemiological studies of depression in children and adolescents	Depression Inventory	1127
8	Thapar, A., Collishaw, S., Pine, D.S., Thapar, A.K. (2012) [1]. Depression in adolescence	Depression Prevalence, Risk and Prevention	889
9	Chorpita, B.F., Yim, L., Moffitt, C., Umemoto, L.A., Francis, S.E. (2000) [35]. Assessment of symptoms of DSM-IV anxiety and depression in children	Depression Inventory	876
10	Saylor, C.F., Finch, A.J., Spirito, A., Bennett, B. (1984) [32]. The Children’s Depression Inventory-A systematic evaluation of psychometric properties	Depression Inventory	817
11	Cyranowski, J.M., Frank, E., Young, E., Shear, M.K. (2000) [43]. Adolescent onset of the gender difference in lifetime rates of major depression	Depression Prevalence	726
12	Emslie, G.J., Rush, A.J., Weinberg, W.A., Kowatch, R.A., Hughes, C.W., Carmody, T., Rintelmann, J. (1997) [44]. A double-blind, randomized, placebo-controlled trial of fluoxetine in children and adolescents with depression	Depression Treatment	708
13	Whittington, C.J., Kendall, T., Fonagy, P., Cottrell, D., Cotgrove, A., Boddington, E. (2004) [45]. Selective serotonin reuptake inhibitors in childhood depression: systematic review of published versus unpublished data	Depression Treatment	696
14	Nanni, V., Uher, R., Danese, A. (2012) [40]. Childhood maltreatment predicts unfavorable course of illness and treatment outcome in depression	Childhood maltreatment and Depression	678
15	Danese, A., Moffitt, T.E., Harrington, H., Milne, B.J., Polanczyk, G., Pariante, C.M., Poulton, R., Caspi, A. (2009) [39]. Adverse childhood experiences and adult risk factors for age-related disease depression, inflammation, and clustering of metabolic risk markers	Adverse Childhood Experiences and Depression	653
16	Widom, C.S., DuMont, K., Czaja, S.J. (2007) [38]. A prospective investigation of major depressive disorder and comorbidity in abused and neglected children grown up	Childhood Abuse and Depressive Disorders	648
17	Twenge, J.M., Nolen-Hoeksema, S. (2002) [36]. Age, gender, race, socioeconomic status, and birth cohort differences on the Children’s Depression Inventory	Depression Inventory	644
18	Smucker, M.R., Craighead, W.E., Craighead, L.W., Green, B.J. (1986) [33]. Normative and reliability data for the Children’s Depression Inventory	Depression Inventory	621
19	Kovacs, M., Feinberg, T.L., Crousenovak, M.A., Paulauskas, S.L., Finkelstein, R. (1984) [46]. Depressive-disorders in childhood	Psychiatric Status and Characteristics	585
20	Costello, E.J., Erkanli, A., Angold, A. (2006) [47]. Is there an epidemic of child or adolescent depression?	Depression Prevalence	575

^a^: citations are based on citations by other documents contained in the WoS index as of 30 August 2021.

Next, VOSviewer software is used to determine the top co-cited documents in the field of childhood and adolescent depression (see Table 4). As mentioned above, because document co-citation analysis allows scholars to dig into documents outside the database, it can provide a useful and complementary perspective for traditional citation analysis. The top co-cited documents listed in Table 4 span a period of four decades. Of these, nine are conceptual studies and five are review articles. The earliest of these documents were authored by Beck [48], Radloff [49], and Kovacs [29]. Beck et al. [48] created the original depression inventory (Beck Depression Inventory (BDI)) to meet the need for a brief self-report measure. Radloff [49] proposed a self-reporting scale for measuring depressive symptoms in the general population-CES-D scale, which was a useful tool for epidemiological research on depression. Kovacs developed a self-reporting childhood and adolescent depression inventory in 1981, which became the most widely used self-reporting measure of depression and underwent thorough psychometric investigation.

There was some overlap with the highly cited documents displayed in Table 3 [29,30,32]. These articles were all produced in the 1980s and related to the child depression scale. However, as mentioned earlier, document co-citation analysis (DCA) confirmed a broader set of influential literature. The co-cited articles listed in Table 4 included not only those about depression, but also methodological-related literature [50]. There are even articles on modeling methods [51,52] and user guides for analytical software [53]. Unsurprisingly, Radloff’s [49] *Applied Psychological Measurement* paper on the CES-D Scale, published over 40 years ago, emerged as the most influential article in the field.

**Table 4:** Twenty most highly co-cited documents, 1970 to 2019.

Rank	Cited Reference	Year	Source	Paper Type	Co-Citations
1	Radloff, L. S. The CES-D Scale [49].	1977	*Applied Psychological Measurement*	Con	1234
2	American Psychiatric Association (APA). Diagnostic and statistical manual of mental disorders.	1994	*Washington DC: American Psychiatric Association*	---	715
3	Kovacs, M., Goldston, D., Obrosky, D.S. Prevalence and predictors of pervasive noncompliance with medical-treatment among youths with insulin-dependent diabetes-mellitus [53].	1992	*Journal of The American Academy of Child and Adolescent Psychiatry*	Emp	493
4	Kaufman, J., et al. Schedule for Affective Disorders and Schizophrenia for School-Age Children Present and Lifetime version (K-SADS-PL) [54].	1997	*Journal of The American Academy of Child and Adolescent Psychiatry*	Emp	460
5	Beck, A.T., et al. An Inventory for Measuring Depression [48].	1961	*Archives of General Psychiatry*	Con	452
6	Hankin, B.L., et al. Development of depression from preadolescence to young adulthood [55].	1998	*Journal of Abnormal Psychology*	Emp	445
7	Baron, R.M., Kenny, D.A. The moderator-mediator variable distinction in social psychological research [50].	1986	*Journal of Personality and Social Psychology*	Con	443
8	Kovacs, M. The Childrens Depression, Inventory [30].	1985	*Psychopharmacology Bulletin*	Con	417
9	Birmaher, B., et al. Childhood and adolescent depression [17].	1996	*Journal of The American Academy of Child and Adolescent Psychiatry*	Rev	352
10	Kovacs, M. Rating-scales to assess depression in school-aged children [29].	1981	*Acta Paedopsychiatrica*	Con	350
11	Hu, L.T., Bentler, P.M. Cutoff criteria for fit indexes in covariance structure analysis [51].	1999	*Structural Equation Modeling*	Con	346
12	Goodman, S.H., Gotlib, I.H. Risk for psychopathology in the children of depressed mothers [56].	1999	*Psychological Review*	Con	345
13	Nolenhoeksema, S., Girgus, J.S. The Emergence of Gender Differences in Depression During Adolescence [16].	1994	*Psychological Bulletin*	Rev	299
14	Lovejoy, M.C., Graczyk, P.A., O’Hare, E., Neuman, G. Maternal depression and parenting behavior [57].	2000	*Clinical Psychology Review*	Rev	290
15	Lewinsohn, P.M., Rohde, P., Seeley, J.R., Fischer, S.A. Age-Cohort Changes in The Lifetime Occurrence of Depression and Other Mental-Disorders [58].	1993	*Journal of Abnormal Psychology*	Emp	289
16	Downey, G. and Coyne, J.C. Children of depressed parents [59].	1990	*Psychological Bulletin*	Rev	287
17	Muthen, L. K., Muthen, B. Mplus User Guide [53].	1998	*---*	---	279
18	West, S.G., Hepworth, J.T. Statistical Issues in The Study of Temporal Data [52].	1991	*Journal of Personality*	Con	267
19	Cox, J.L., Holden, J.M., Sagovsky, R. Detection of postnatal depression [60].	1987	*British Journal of Psychiatry*	Con	263
20	Saylor, C.F., et al. The Childrens Depression Inventory [32].	1984	*Journal of Consulting and Clinical Psychology*	Rev	254

Note. Con = conceptual; Emp = empirical; Rev = review. ^a^ Another two versions of the APA DSM received 429 and 333 co-citations respectively in 2000 and 1987 [61,62].

### 3.3. Research Topics

Keywords reflect the core content of the article, so keyword analysis can help identify important topics in a research field. Keyword co-occurrence analysis is based on the keywords contained in the article, and therefore can provide a more detailed picture of the composition of the knowledge base than co-citation analysis. Like duplicate author processing, keyword co-occurrence analysis must also deal with keywords with the same meaning, like “mother” and “mothers”, or “mental-health” and “mental health.” The threshold of co-occurrences of a keyword was set to 50, and 257 co-occurring keywords were selected for display.

**Figure 4:**
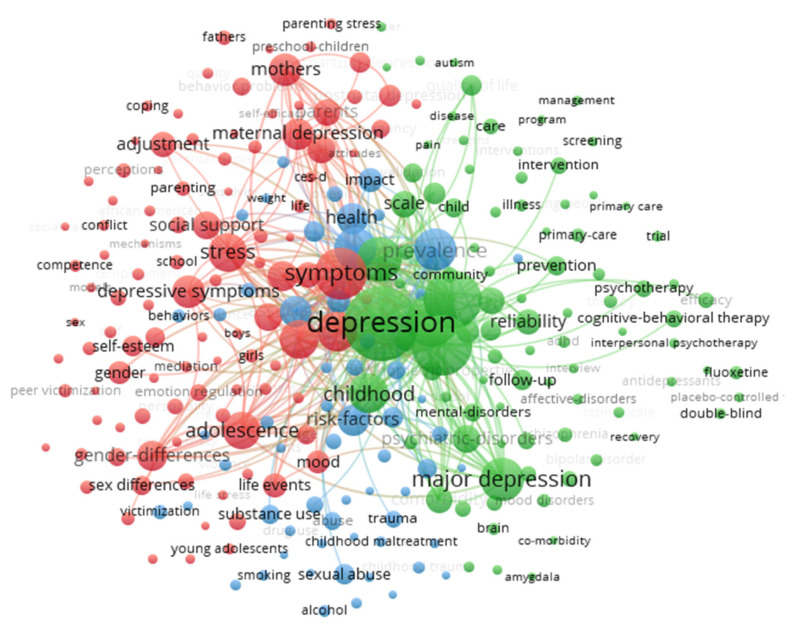
Keyword co-occurrence map from 1970 to 2019 (threshold 50 co-occurrences, display 257 keywords).

In the keyword co-occurrence network diagram, common keywords are represented by larger circles and character fonts, and the lines between keywords reflect the correlation between them. As shown in Figure 4, all keywords displayed are grouped into three clusters of different colors. Accordingly, the themes of childhood and adolescent depression research can be divided into the following categories:

(1) Childhood and adolescent depression symptoms and prevalence (green) contains keywords such as “depression”, “major depression”, “symptoms”, “disorder”, “anxiety”, “children”, “prevalence”, “comorbidity”, “scale”, and “inventory”. The location of this cluster in the center of the figure emphasizes its status as a cognitive structure in the field of childhood depression. Studies on evaluating the symptoms of depression in children have observed prevalence rates of, for example, 2% in Iran [63], 3% in India [64], 4% in Turkey [65], 4% in Greece [66], 5% in America [67], 10% in Italy [68], and 20% in China [69,70]. Although results may be biased due to differences in sample size and measurement instruments, the consensus among most researchers is that the prevalence of depression is substantial in children and adolescents [71,72]. Some scholars have found that depressive symptoms in children and adolescents are correlated with age, gender, and socioeconomic level [63,73,74,75]. In addition, some scholars have also explored whether depression symptoms are stable in children; for example, Edelsohn et al. [76] found that reports of depressive symptoms in children were relatively stable during an interval of 4 months. The results of Ialongo [77] and Dubois [78] showed that children’s depressive symptoms have much longer-term stability.

Measurement of anxiety and depression among young people has long been a priority in both clinical and research contexts. Accordingly, researchers have developed a number of evaluation instruments to measure depression in children and adolescents. Table 5 outlines the ones most commonly used in scientific literature. Additionally, some researchers have discussed the reliability and validity of different national versions of the child depression scale, such as the Japanese version of the Children’s Depression Inventory [79], the German version of the Revised Children’s Anxiety and Depression Scale [80], and the Basque version of the Children’s Depression Scale [81].

(2) Parental depression and child behavioral or emotional problems (red) covers “mothers”, “maternal depression”, “parents”, “fathers”, “family”, “parental depression”, “parenting stress”, “caregivers”, “pregnancy”, and “postnatal depression”. One set of themes relates to parental depression and child mental health and behavioral problems.

Associations between parental depression and child affective and destructive disorders have been fully demonstrated [104]. Research has shown that children with parents suffering from major depression (MDD) are 4–6 times more likely to develop MDD than other children [105]. However, scholars have not reached a consensus on the extent to which parental depression affects depression in children. Some studies have highlighted that maternal mental illness is a potential risk factor for childhood psychiatric disorders [106], while paternal depression is not associated with an increased risk of adolescent psychopathology [107]. However, the findings of some scholars indicate that the negative interpersonal relationship effects of parental depression on child psychopathology may not be limited to mothers [108,109]. They emphasized that fathers may play a role in reducing or exacerbating the long-term adverse effects of maternal depression on child behavior problems [110]. Studies have shown that children with depressed mothers exhibit more behavioral problems than children whose mothers are not depressed [111] and that maternal depression is negatively associated with social competence in children and positively associated with behavioral problems [112]. Additionally, some studies have explored the association between maternal depression and children’s outcomes in terms of economic resources, maternal parenting behavior, family environment and genetic factors [113,114].

Another aspect of this cluster mainly concerns the negative impact of postpartum depression on child development and well-being [115,116]. There have been some interesting studies; for example, depression in fathers in the postnatal period is associated with later psychiatric disorders in their children, independently of maternal postnatal depression [117]. Another example is that female fetuses are more susceptible to the mother’s stress responses during pregnancy, which continues into adolescence [118]. All of these aspects could be important for future public health interventions.

(3) Childhood abuse and depression (blue) are represented by keywords like “sexual abuse”, “abuse”, “maltreatment”, “experiences”, “child abuse”, “childhood trauma”, “physical abuse”, and “stressful life events”. Childhood trauma (CT) is very common and may have long-term consequences for physical and mental health, especially depression [119,120]. Studies in this cluster have shown that: (1) A history of childhood abuse and neglect can exacerbate the psychosocial dysfunction associated with depression [121], and even induce new or recurring depressive episodes [122]; meanwhile, childhood maltreatment not only increases lifetime risk of depression, but also has a negative impact on clinically relevant measures of depression. Therefore, early recognition of childhood abuse and appropriate intervention can play an important role in preventing depression [37,40]. (2) Taking age and gender into account, existing findings suggest that the effects of childhood maltreatment on depression may increase with age, and that the effects at different developmental stages may lead to distinct psychiatric symptoms in adulthood [123]. Some research has shown that the association between childhood abuse and depression is most important for women, while other studies have found that men and women who report a history of childhood abuse are equally likely to suffer from severe depression in adulthood [124,125]. (3) Existing research has investigated possible moderating or mediating effects on the association between childhood maltreatment and depressive and anxiety disorders such as emotion dysregulation [126,127], as well as resilience [128,129], personality traits [130,131,132,133], maternal relationship quality and peer social acceptance [134], feelings of shame [135], perceived friendship (social support from family and friends) [136], rumination [137], self-compassion and gratitude [138] and adult negative life events [139].

**Figure 5:**
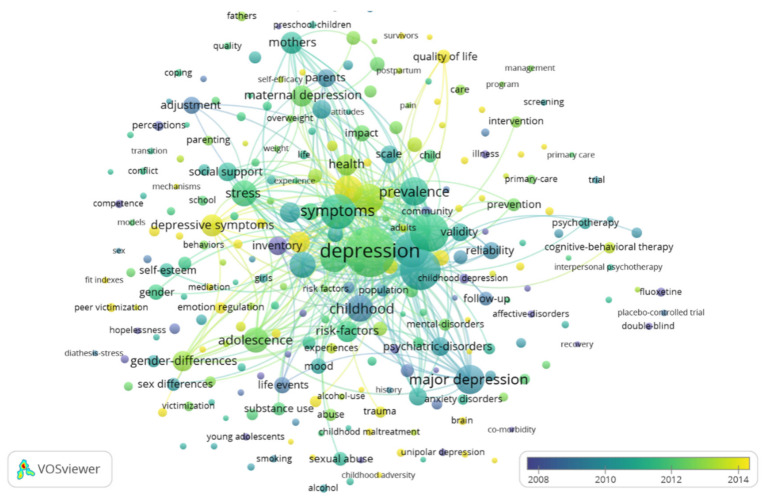
Distribution of keywords based on the time of appearance. Keywords are marked by circles in different colors (blue early; yellow later; threshold 50 co-occurrences, display 257 keywords).

Keywords can also serve as indicators to reflect the changes of research hotspots over time, which can help researchers predict the “research front” of a knowledge base and quickly discover the latest topical trends in the literature. The VOSviewer can apply colors to keywords based on when they appear in the journal. As shown in Figure 5, the yellow nodes in the figure represent the topics more recently highlighted in the literature.

Childhood trauma, abuse, and other adverse experiences are important early social risk factors for the development of depression. Yet, the pathway through which exposure to adverse childhood experiences affects the prevalence and severity of depressive symptoms remains largely unclear. Recently, scholars have studied the relationship between adverse childhood experiences and depression, especially in terms of the mediating or moderating effects of resilience or emotion regulation. These relatively new studies have yielded some useful results. For example, children high in emotion dysregulation may be at increased risk for depression [140,141]. They underscored the importance of assessing emotion regulation abilities in abused youth and noted that interventions targeting emotional regulation strategies may help reduce depression among children suffering from adversity [142,143]. They also found that resilience may play an important role in the relationship between adverse childhood experiences and depressive symptoms, so improving resilience may offer a new possibility for preventing adolescent depression [128].

## 4. Discussion and Conclusions

The amount of literature on childhood and adolescent depression has become substantial since its beginnings in the 1970s. The significant increase in the number of documents allows us to see scholars’ increasing attention and continuous thinking about childhood and adolescent depression. Our topographical analysis of the literature found a skewed geographical distribution with a majority of the literature coming from the United States, England, Canada, Australia, and Netherlands. Fortunately, this imbalance has eased over the past decade. In particular, we have seen an increasing proportion of papers being produced by scholars in Asia and Latin America. Nevertheless, as shown in Figure 2, there are still many ‘blank areas’ on the world map, representing that there is little or no research and practice on childhood and adolescent depression in these areas. Most of these blank spots are in African countries, which means that the study of child depression in these areas is urgent. Therefore, we advocate for greater geographic diversity in the sources of literature on childhood and adolescent depression.

The citation analysis in Table 3 and Table 4 highlights the important role of bibliometrics in the analysis of the development of knowledge in a particular area. Judging from the results of this article, it is feasible to trace the evolution of childhood and adolescent depression research through citation analysis and co-citation analysis. It is worth noting that these articles not only include literature centered on childhood and adolescent depression [15,17,29,30,56,91] but also related articles that provide a conceptual basis for the knowledge base for childhood and adolescent depression [48,49,50]. We assert that the identification of these knowledge bases through co-citation analysis is not only a recognition of scientific mapping methods, but also high-quality scholarship.

Another contribution of this paper lies in the empirical identification of research topics about childhood and adolescent depression relying on keyword co-occurrence analysis. These topics basically summarize three major aspects of childhood and adolescent depression research, including child and adolescent depression symptoms and prevalence, parental depression and child behavioral or emotional problems, and childhood abuse and depression. Although many diagnostic instruments for depression have been developed and validated by researchers, some of the instruments used in existing studies have demonstrated certain weaknesses in their validity or reliability. Therefore, a simple, manageable, effective, and reliable instrument that can diagnose and measure the severity of symptoms of depression in children and adolescents is needed in the future. The prevention of depression is a focus of the World Health Organization [144]. However, current depression prevention programs are mainly targeted at adolescents, and little attention is paid to children under 10 years old. Meanwhile, depression treatment is also important. The World Health Organization and the World Bank claimed in 2016 that the annual cost of depression and other similar diseases to the global economy had reached one trillion US dollars. A series of studies on this subject has also been carried out recently. Researchers are eager to find tools or preventative intervention strategies that can reduce or alleviate childhood and adolescent depression. This study has found that the two keywords “emotion regulation” and “resilience” have become research frontiers in recent years. Therefore, future research should be carried out in these areas. Furthermore, earlier identification and treatment, attention to the need for treatment of parental depression, and the development of new therapeutics for adolescents who do not have a response to current treatments would be beneficial.

The future research potential of sanitation may be informed by our analysis. First, as the global average temperature has risen, climate warming has caused a series of impacts on human health. Many studies focus on the relationship between temperature and people’s mental health, but most of these studies focus on the general population or the elderly [145,146,147], and there is a lack of relevant studies on children and adolescents. Secondly, breakthroughs in research methods and analytical frameworks can be sought. In terms of research methodology, innovative methods such as machine learning and big data mining can be used in depression research. In view of the continuous optimization of machine learning algorithms and the increasing information available from big data, the application of machine learning to modeling, especially the establishment of interdisciplinary models, will provide important ideas for depression research in children and adolescents.

## Figures and Tables

**Table 1:** Top 10 most active journals publishing on childhood and adolescent depression.

Journal Title	Number of Publications	IF ^a^	H-Index	Subject Category of the Journal (2020)
Journal of Affective Disorders	352	5.515	59	Clinical Neurology (Q2) Psychiatry (Q1)
Journal of The American Academy of Child and Adolescent Psychiatry	299	10.606	95	Pediatrics (Q1) Psychiatry (Q1) Psychology, Developmental (Q1)
Journal of Abnormal Child Psychology	240	4.836	67	Psychology, Clinical (Q2) Psychology, Developmental (Q1)
Journal of Youth and Adolescence	163	5.613	45	Psychology, Developmental (Q1)
Journal of Clinical Child and Adolescent Psychology	146	5.644	45	Psychology, Clinical (Q1) Psychology, Developmental (Q1)
Journal of Adolescence	120	4.181	39	Psychology, Developmental (Q2)
Journal of Child Psychology and Psychiatry	118	9.917	55	Psychiatry (Q1) Psychology (Q1) Psychology, Developmental (Q1)
Development and Psychopathology	111	5.643	40	Psychology, Developmental (Q1)
Journal of Child and Family Studies	110	2.91	18	Family Studies (Q2) Psychiatry (Q3) Psychology, Developmental (Q3)
Journal of Abnormal Psychology	100	8.756	60	Psychiatry (Q1) Psychology Clinical (Q1)

Note: IF ^a^: Five-year impact factor, impact factor data from the 2020 edition of Journal Citation Reports^®^ in Web of Science.

**Table 2:** Ranking of the 20 most highly cited authors, 1970 to 2019.

Author	Country/Institute	Active	Citations ^a^	Numbers	Average Citations Per Publication	H-Index
Seeley, JR	USA/Oregon Research Institute	1991–2014	8258	50	165.2	40
Kovacs, M	USA/University of Pittsburgh	1981–2019	8064	47	171.77	33
Lewinsohn, PM	USA/Oregon Research Institute	1990–2014	7864	51	172.06	40
Birmaher, B	USA/University of Pittsburgh	1992–2018	6434	70	91.94	44
Ryan, ND	USA/University of Pittsburgh	1986–2019	4945	56	88.32	38
Pine, DS	USA/National Institute of Mental Health (NIMH)	1998–2019	3939	20	196.95	18
Emslie, GJ	USA/University of Texas Southwestern Medical Center Dallas	1987–2018	3862	54	71.56	31
Hammen, C	USA/University of California Los Angeles	1984–2016	3623	36	100.72	31
Brent, DA	USA/University of Pittsburgh	1987–2018	3619	42	86.31	32
Garber, J	USA/Vanderbilt University	1980–2019	3603	44	82.02	28
Cole, DA	USA/Vanderbilt University	1986–2019	3527	49	71.98	33
Klein, DN	USA/State University of New York Stony Brook	1994–2019	3327	48	69.33	27
Dahl, RE	USA/University of California Berkeley	1990–2019	3109	41	75.83	28
Weissman, MM	USA/Columbia University	1980–2019	3005	26	115.58	21
Wagner, KD	USA/University of Texas Medical Branch Galveston	1993–2014	2902	35	86.46	23
Hankin, BL	USA/University of Illinois Urbana-Champaign	2001–2019	2775	37	72.92	24
Asarnow, JR	USA/University of California Los Angeles	1985–2019	2757	43	64.98	26
McCauley, E	USA/University of Washington	1988–2017	2625	42	62.50	27
Thapar, A	England/Cardiff University	1994–2019	2257	38	60.68	20
Compas, BE	USA/Vanderbilt University	1993–2019	2229	32	67.88	19

^a^: citations are based on citations by other documents contained in the WoS index as of 30 August 2021.

**Table 5:** Summary of the main instruments used in scientific literature.

Name	Simple Description	References
State-Trait Anxiety Inventory for Children (STAIC)	A widely used instrument of 20-item instrument to measure anxiety symptoms.	Speilberger, 1973 [82]
Children’s Depression Scale (CDS)	A commonly used instrument of 66-item instrument to measure child depression in psycho-educational and clinical spheres.	Lang and Tisher, 1978 [83]
Children’s Depression Rating Scale-Revised (CDRS-R)	A 17-item instrument designed to measure severity of depression in children aged 6 to 12 years. Total score ranges from 17 to 113. 40 or more presents a major depressive disorder.	Poznanski et al., 1979, 1984, 1985 [13,84,85]
Peer Nomination Inventory of Depression (PNID)	A well-developed peer-report instrument with 19 items rating depression, happiness, and popularity	Lefkowitz & Tesiny, 1980 [86]
Center for Epidemiological Studies Depression Scale for Children (CES-DC)	A 20-item rating scale ranging from 0 to 3, a general measure of current childhood psychopathology	Weissman et al., 1980 [87]
Depression Self-Rating Scale for Children (DSRSC)	An 18-item self-report questionnaire used to measure the depression status of the children.	Birleson, 1981 [88]
Child Behavior Checklist (CBCL)	A caregiver-completed and widely utilized questionnaire consists of 20 social competence items and 118 behavior problems items.	Achenbach & Edelbrock, 1981 [89]
Child Behavior Checklist-Teacher Report Form (CBCL-T)	A 120-item standardized teacher rating scale assessing social competence and behavior problems in children and adolescents	Achenbach & Edelbrock, 1986 [90]
Children’s Depression Inventory (CDI)	A widely used self-report measure of 27-item measure designed for school-age children and adolescents. Total score ranges from 0 to 54.	Kovacs, 1985, 1992 [30,91]
Revised Children’s Manifest Anxiety Scale (RCMAS)	A 37-item self-report measure that provides an estimate of a child’s level and degree of experienced anxiety.	Reynolds & Richmond, 1985 [92]
Reynolds Adolescent Depression Scale (RADS)	A 30-item scale used to assess the severity of depressive symptomatology in adolescent populations.	Reynolds, 1986 [93]
Reynolds Child Depression Scale (RCDS)	A 30-item scale designed to assess depressive symptomatology in children between the ages of 8 and 12 years.	Reynolds, 1989 [94]
Short Mood and Feelings Questionnaire (SMFQ)	A brief, easy-to-administer, self-report measure of childhood and adolescent depression	Angold et al., 1995a [34]
Child and Adolescent Psychiatric Assessment (CAPA)	A semistructured interview designed for use by clinicians or highly trained lay persons to assess 9-to 17-year-olds.	Angold et al., 1995b [95]; Angold et al., 2000 [96]
Spence Children’s Anxiety Scale (SCAS)	A 45-item scale with 38 items designed to assess children’s report of anxiety symptoms and 7 items designed to assess social desirability.	Spence, 1997 [97]
Schedule for Affective Disorders and Schizophrenia for School-age Children-Present and Lifetime version (K-SADS-PL)	An interviewer-based schedule designed for assessing and diagnosing episodes of psychopathology in youngsters.	Kaufman et al., 1997 [54]
Positive and Negative Affect Schedule for Children (PANAS-C)	A 20-item self-report measure consisting of two scales: Positive Affect (PA) and Negative Affect (NA).	Crook et al., 1998 [98]
Diagnostic Interview for Children and Adolescents (DICA)	A respondent-based interview schedule for youngsters aged 6 to 17 years that can be administered either by clinicians or by lay interviewers.	Reich, 2000 [99]
Diagnostic Interview Schedule for Children (DISC-IV)	An instrument designed to address more than 30 psychiatric diagnoses that occur in children and adolescents	Shaffer et al., 2000 [100]
Revised Child Anxiety and Depression Scales (RCADS)	A 47-item self-report questionnaire designed to assess for clinical syndromes in youth.	Chorpita et al., 2000 [35]
Kutcher Adolescent Depression Scale (KADS)	An 11-item, self-report instrument designed to aid in diagnosis and monitoring the change in the severity of symptoms during the course of treatment.	Brooks & Kutcher, 2001 [101]
Children’s Thought Questionnaire (CTQ)	A 60-item scale designed to assess children’s self-reported anxious, depressive, and positive thoughts.	Marien and Bell, 2004 [102]
Dysfunctional Attitudes Scale for Children (DAS-C)	A 22-item self-report measure with sound psychometric properties.	D’Alessandro and Burton, 2006 [103]

## Data Availability

Publicly available datasets were analyzed in this study. This data can be found here: [http://webofscience.com].
